# 36 Physical activity and sedentary behaviour among Slovenian police officers

**DOI:** 10.1093/eurpub/ckae114.095

**Published:** 2024-09-26

**Authors:** Nastja Podrekar Loredan, Kaja Kastelic, Jure Žitnik, Nejc Šarabon

**Affiliations:** University of Primorska, Faculty of Health Sciences, Izola, Slovenia; Human Health in the Built Environment, InnoRenew CoE, Izola, Slovenia; Department of Health Study, Andrej Marušič Institute, University of Primorska, Koper, Slovenia; University of Primorska, Faculty of Health Sciences, Izola, Slovenia; University of Primorska, Faculty of Health Sciences, Izola, Slovenia; Human Health in the Built Environment, InnoRenew CoE, Izola, Slovenia

## Abstract

**Purpose:**

Police officers (PO) are exposed to several health-related risk factors including shift work, extended work schedules, dangerous, and traumatic events. Although police work is often seen as active, PO spend the majority of worktime sitting in offices or driving cars. The prevalence of sedentary behaviour (SB) and physical activity (PA) among Slovenian PO is obscure. Therefore, the aim of this project was to assess the level of PA and SB among Slovenian PO and to design a health promotion programme.

**Methods:**

The online Sleep, Sedentary Behaviour and Physical Activity Questionnaire (DABQ) was sent via e-mail to 1,500 members of the police union. 480 PO (age 46.8 ± 8.1 years, 75% males) completed the questionnaire, out of which 66 PO (age 41.7 ± 7.2 years, 74% males) agreed to participate in the objective assessment of SB and PA and wore activPAL and ActiGraph accelerometers for a period of ten days. Based on the findings the health promotion programme was developed, focusing on reducing SB, promoting PA and emphasizing the importance of workplace ergonomics. It consisted of educational part, workshops and development of a mobile application for enhancing daily PA.

**Results:**

The results of the questionnaire indicate that 62% of PO achieve World Health Organisation recommendations for weekly PA, 34% perform separate strength and power training. However, PO spent 9.7 ± 3.5 h/day sedentary, out of which 7.4 ± 2.6 h during worktime. Only 7% of PO use active transport. The results of the objective assessment suggest that the prevalence of SB among PO is 10.5 ± 1.5 h/day, time spent in low-intensity PA 4.9 ± 1.2 h/day and 1.3 ± 0.5 h/day in moderate to vigorous PA. The health promotion programme that followed the assessments was very well received among PO.

**Conclusions:**

The results suggest high levels of SB among Slovenian PO, especially during worktime. On the other hand, more than half of PO meet current recommendations for weekly PA. Based on the results and feedback from the participating PO, the implementation of health promotion programmes in police forces is strongly recommended on national level.

